# Temporal factors affecting somatosensory–auditory interactions in speech processing

**DOI:** 10.3389/fpsyg.2014.01198

**Published:** 2014-11-04

**Authors:** Takayuki Ito, Vincent L. Gracco, David J. Ostry

**Affiliations:** ^1^Haskins Laboratories, New Haven, CT, USA; ^2^McGill University, Montréal, QC, Canada

**Keywords:** facial skin sensation, speech perception, speech production, electroencephalography, event-related potentials

## Abstract

Speech perception is known to rely on both auditory and visual information. However, sound-specific somatosensory input has been shown also to influence speech perceptual processing (Ito et al., 2009). In the present study, we addressed further the relationship between somatosensory information and speech perceptual processing by addressing the hypothesis that the temporal relationship between orofacial movement and sound processing contributes to somatosensory–auditory interaction in speech perception. We examined the changes in event-related potentials (ERPs) in response to multisensory synchronous (simultaneous) and asynchronous (90 ms lag and lead) somatosensory and auditory stimulation compared to individual unisensory auditory and somatosensory stimulation alone. We used a robotic device to apply facial skin somatosensory deformations that were similar in timing and duration to those experienced in speech production. Following synchronous multisensory stimulation the amplitude of the ERP was reliably different from the two unisensory potentials. More importantly, the magnitude of the ERP difference varied as a function of the relative timing of the somatosensory–auditory stimulation. Event-related activity change due to stimulus timing was seen between 160 and 220 ms following somatosensory onset, mostly around the parietal area. The results demonstrate a dynamic modulation of somatosensory–auditory convergence and suggest the contribution of somatosensory information for speech processing process is dependent on the specific temporal order of sensory inputs in speech production.

## INTRODUCTION

Multiple sensory inputs seamlessly interact in the process of speech perception. Information from a talker comes to a listener by way of the visual and auditory systems. The McGurk effect (McGurk and MacDonald, 1976) is a compelling demonstration of how the influence of audio–visual (AV) information is used in speech perceptual processing. Electrophysiological (Giard and Peronnet, 1999; Foxe et al., 2000; Molholm et al., 2002) and functional imaging studies (Macaluso et al., 2000; Calvert et al., 2001; Foxe and Simpson, 2002) have provided evidence that cortical multisensory integration can occur at early stages of cortical processing. In addition, evidence for multisensory AV processing has been identified over left central scalp which has been hypothesized to reflect sensorimotor integration (Molholm et al., 2002).

In contrast to the main focus of AV interactions, recent findings of an orofacial somatosensory influence on the perception of speech sounds suggest a potential role for the somatosensory system in speech processing. For example, air puffs to the cheek that coincide with auditory speech stimuli alter participants’ perceptual judgments (Gick and Derrick, 2009). In addition, precise orofacial stretch applied to the facial skin while people listen to words, alters the sounds they hear as long as the stimulation applied to the facial skin is similar to the stimulation that normally accompanies speech production (Ito et al., 2009). Whereas these and other psychophysics experiments have examined somatosensory–auditory interactions during speech processing in behavioral terms (Fowler and Dekle, 1991), neuroimaging studies exploring the relation between multisensory inputs have been limited to AV interaction (van Atteveldt et al., 2007; Pilling, 2009; Vroomen and Stekelenburg, 2010; Liu et al., 2011).

The temporal relationship between multiple sensory inputs is one important factor for the tuning of multi-sensory interactions (Vroomen and Keetels, 2010). At a behavioral level, multiple sensory inputs are not required to arrive exactly at the same time, but some level of temporal proximity is needed to induce an interaction. In AV speech, the visual inputs influence speech perception even when the visual input leads the auditory input by as much as 200 ms (Munhall et al., 1996; van Wassenhove et al., 2007). Temporal relationships have also been examined in somatosensory–auditory interactions (see review for Occelli et al., 2011), but only in temporal perception of non-speech processing. In speech production, the temporal relationship between somatosensory inputs associated with articulatory motion and their acoustic consequences varies. For the most part, somatosensory inputs due to articulatory motion occur in advance of acoustic output (Mooshammer et al., 2012). If the influence of somatosensation on speech perception is based on the temporal mapping between somatosensory and auditory inputs that is acquired in speech production, it is plausible then that cortical potentials may be influenced in response to the specific timing of somatosensory–auditory interactions during speech processing. Moreover, the contribution of auditory–somatosensory interactions during speech processing using speech-production-like patterns of facial skin stretch, may yield important insight into the link between speech production and perception.

In the current study, we investigate auditory and somatosensory interactions during speech processing using event-related potentials (ERPs). A robotic device was used to generate patterns of facial skin deformation similar in timing and duration to those experienced during speech production, which induces an interaction with speech sound processing (Ito et al., 2009; Ito and Ostry, 2012). We observed somatosensory–auditory interactions during speech sound processing as well as a dynamic modulation of the effects of multisensory input as a result of relative timing differences between the two sensory stimuli. ERPs using electroencephalography (EEG) have benefits for the investigation of temporal asynchronies because of their better temporal resolution in comparison with the other brain imaging techniques. The findings reveal neural correlates of multisensory temporal coding and a dynamic modulation of multisensory interaction during speech processing. The results have implications for understanding the integral link between speech production and speech perception.

## MATERIALS AND METHODS

### PARTICIPANTS

Eighteen native speakers of American English participated in the experiment (12 for ERP recording and 6 for the separate behavioral control test). The participants were all healthy young adults with normal hearing and all reported to be right-handed. All participants signed informed consent forms approved by the Yale University Human Investigation Committee.

### EXPERIMENTAL STIMULATION AND TASK

We examined interaction effects between speech sound processing and orofacial somatosensory processing. ERPs were recorded in response to either individual or paired somatosensory and auditory stimulation. The somatosensory and auditory pairs used in the current study have been found previously to induce perceptual modulation in speech sound perception (Ito et al., 2009) and somatosensory judgment (Ito and Ostry, 2012).

A small robotic device (SenSable Technology, Phantom 1.0) applied skin stretch loads for the purpose of orofacial somatosensory stimulation. The details of the somatosensory stimulation device have been described in our previous studies (Ito et al., 2009; Ito and Ostry, 2010). Briefly, two small plastic tabs were attached bilaterally with tape to the skin at the sides of the mouth. The tabs were connected to the robotic device using monofilament and skin stretch was applied in an upward direction. Skin stretch consisted of a single cycle of a 3-Hz sinusoid with 4 N maximum force. This temporal pattern has successfully induced somatosensory ERPs in a previous study (Ito et al., 2013).

Audio stimulation was delivered binaurally through plastic tubes (24 cm) and earpieces (Etymotic Research, ER3A). We used a single synthesized speech utterance that was midway in a 10-step sound continuum between “*head*” and “*had.*” The speech continuum was created by shifting the first (F1) and the second (F2) formant frequencies in equal steps (Purcell and Munhall, 2006). The original sample sounds of “*head*” and “*had*” were produced by a male native speaker of English. These same sounds were used in a previous study demonstrating modulation of speech perception in response to facial skin stretch (Ito et al., 2009). We chose the center point of the continuum as an example of a perceptually ambiguous sound. In the current study, participants reported 68.5% of stimuli as “*head*” due to the ambiguity of the stimulus.

**FIGURE 1:**
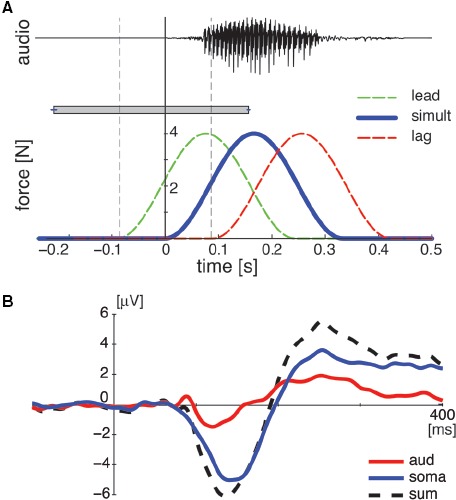
**(A)** Temporal relationship in paired somatosensory–auditory stimulation. The top panel represents acoustic signal for auditory stimulation. The bottom panel represents the temporal force pattern of facial skin stretch for somatosensory stimulation. The horizontal rectangle with error bars represents the temporal range of somatosensory onset relative to auditory onset over which participants perceived somatosensory and auditory stimuli as simultaneous for the behavioral control study. **(B)** A representative example of two uni-sensory responses (aud and soma) and the sum response at Cz for the simultaneous condition. Each line represents the data averaged across all subjects.

We used three somatosensory–auditory conditions that varied according to the time lag between the stimuli. The variations were 90 ms lead, simultaneous, and 90 ms lag of the somatosensory onset relative to the auditory onset. A 90-ms temporal asynchrony was chosen because a 90-ms somatosensory lead reliably modulated speech perception in a previous study (Ito et al., 2009). Figure 1A shows three temporal relationships between somatosensory and auditory stimuli (lead, lag, and simultaneous). Two unisensory conditions (somatosensory alone and auditory alone) were also assessed. The stimulus condition was changed every trial in random order. The inter-trial interval was varied between 1000 and 2000 ms after each response in order to avoid anticipation and habituation.

The participant’s task was to indicate whether the sound they heard was “*head*” or not. The participants’ response was recorded by key press. In the somatosensory alone condition, the participants were instructed to answer not “*head*” since there was no auditory stimulation. Participant judgments and the reaction time from the onset of the stimulus to the key press constituted the behavioral measures. The participants fixated their gaze on a cross without blinking in order to eliminate artifacts during ERP recording. The cross was removed every 10 trials and the participants were given a short break.

### EEG ACQUISITION AND DATA PROCESSING

#### RECORDING AND PRE-PROCESSING

Event-related potentials were recorded from 64 electrodes (Biosemi ActiveTwo) in response to five stimulus conditions: somatosensory stimulation alone (soma), auditory stimulation alone (aud), and paired somatosensory and auditory stimulation (pair: lead, simultaneous, lag). Hundred ERPs per condition were recorded. Trials with blinks and eye movement were rejected offline on the basis of horizontal and vertical electro-oculography (over ±150 µV). More than 85% of trials per condition were included in the analysis. EEG signals were filtered with a 0.5–50 Hz band-pass filter and re-referenced to the average across all electrodes. The effect of temporal manipulation was analyzed in two ways: somatosensory and auditory viewpoint depending on the alignment of the data at either the somatosensory or auditory onset. In both analyses, a single epoch was extracted in the range between -500 and 1000 ms relative to either somatosensory or auditory stimulus onset. Bias levels were adjusted using the average amplitude in the pre-stimulus interval (-200 to -100 ms).

#### SOMATOSENSORY ANALYSIS

The ERPs in the “pair” condition were aligned at the somatosensory onset. Neural response interactions were identified by comparing ERPs obtained using somatosensory–auditory stimulus pairs with the algebraic sum of ERPs to the unisensory stimuli presented separately by following the method applied in previous studies of somatosensory–auditory interactions (Foxe et al., 2000; Murray et al., 2005). The “sum” ERPs were calculated by summing auditory-alone potentials (aud) with an appropriate temporal shift (either 90 ms lead, 0 or 90 ms lag) with somatosensory-alone potentials (soma; see Figure 1B as representative example of the aud, soma, and sum ERP in the simultaneous condition). This analysis is based on the assumption that the summed ERP responses from the unisensory conditions should be equivalent to the ERP from the same stimuli presented simultaneously, if neural responses to each of the unisensory stimuli are independent (Calvert et al., 2001). Accordingly, divergence between the “sum” ERPs from the two unisensory conditions and “pair” ERPs from paired somatosensory–auditory conditions indicates non-linear interaction between the neural responses to the multisensory stimuli. Note that this approach is limited in non-linear multisensory interaction and is not sensitive to linear multisensory convergence wherein processes to two sensory modalities might interact, but not show any additional activation in electro cortical potentials.

We used the global field power (GFP) to compare the “pair” and “sum” ERPs to identify general changes in electric field strength over the entire head. GFP is the root mean square computed over the average-referenced electrode values at a given instant in time (Lehmann and Skrandies, 1980; Murray et al., 2008). GFP is equivalent to the spatial standard deviation of the scalp electric field, and yields larger values for stronger fields. The use of a global measure was in part motivated by the desire to minimize observer bias that can follow from analyses restricted to specific selected electrodes. We determined GFP amplitude using a temporal window that shows the changes in this measure over the course of the response. The corresponding temporal intervals were determined based on our observation across the three “pair” conditions described in Section “Results.” ERP comparisons at the representative electrodes (Fz, Cz, and Pz) follow. These electrodes were chosen in order to sample the whole map, irrespective of asymmetry and minimizing the number of comparisons.

A 60-ms time window was chosen for the statistical analysis of the GFP amplitude and of the ERP amplitude at the representative electrodes (Fz, Cz, and Pz). In the GFP analysis, we used repeated measures two-way ANOVA to test for differences related to the relative timing of the responses for the somatosensory and auditory stimulation (90 ms lead and lag, and simultaneous) and for the difference between the “sum” of the two unisensory ERPs and “pair” somatosensory–auditory ERP. We also applied repeated measures three-way ANOVA to three electrodes (Fz, Cz, and Pz).

We also compared the topographic map differences between “sum” and “pair” ERPs across the three stimulus timing conditions (lead, lag, and simultaneous). A difference in amplitude was obtained by subtracting “sum” ERPs from “pair” ERPs at each electrode. As an index of topographic difference between the two electric fields, a global dissimilarity measures (DISS) was used (Lehmann and Skrandies, 1980). This parameter is computed as the square root of the mean of the squared difference between the potentials measured at each electrode (versus the average reference), each of which is first scaled to unitary strength by dividing by the instantaneous GFP. This value can range from 0 to 2, where 0 indicates topographic homogeneity and 2 indicates topographic inversion (Murray et al., 2008).

#### AUDITORY ANALYSIS

The ERPs in the “pair” condition were aligned at auditory onset. We reconstructed auditory-like potentials by subtracting somatosensory potentials (soma) from the “pair” potentials at the corresponding temporal shift in each condition, instead of applying the sum of two uni-sensory conditions as done in the somatosensory analysis. Our rationale is that since the mechanism of auditory ERPs in speech processing is well established (e.g., Näätänen and Picton, 1987; Martin et al., 2008 for review), comparing auditory-like potentials in the “pair” condition with the typical auditory ERP (aud) is a way to evaluate the potential multisensory interaction effect. As in the analyses using the algebraic sum described above, we expected that “pair” ERPs with the removal of the somatosensory potentials would be equivalent to the auditory-alone ERP, if neural responses to each of the unisensory stimuli are independent. The subtracted potential should be different from the auditory responses if there is a non-linear interaction. The ERP results were also compared with participants’ behavioral performance, that is, the probability that the stimulus was identified as “head” during the test as mentioned later.

We focused on the first negative peak (N1) and the following positive peak (P2) at Fz and Cz because as a general tendency the maximum amplitude of the auditory ERP is observed at these electrodes and this was true of the current responses. Note that the negative peak and positive peak do not mean negative or positive value but the direction of electrical deviation, and hence N1 can be a positive value as long as it is going in a negative direction (i.e., Ostroff et al., 1998). A 60-ms time window was used to calculate the response amplitude. The analysis window was centered at the ERP peak location for each participant and each condition. The peaks associated with N1 and P2 were identified in the time periods (100–200 ms for N1 and 200–300 ms for P2) following stimulus onset. Repeated measures ANOVA was applied to assess differences in the four conditions (three “pair” potentials and one auditory potential). Pairwise comparisons with Bonferroni correction followed.

### BEHAVIORAL PERFORMANCE

Behavioral performance was evaluated using reaction time and judgment probability separately. Reaction time was calculated as the period between auditory onset and the behavioral response (key press for the speech sound identification). Repeated measures ANOVA was used to assess differences in reaction time across five conditions: three “pair” and two unisensory conditions. We also calculated the probability that the participant classified the sound as “head.” The somatosensory alone condition was not included in this analysis. Note that in more than 95% of somatosensory trials participants responded not “head” as instructed. Repeated measures ANOVA was used to compare judgment measures across conditions.

We also examined the extent to which the perceptual judgments were correlated with ERP amplitude change that were observed in response to changes in the relative timing of somatosensory–auditory stimulation. The correlation analysis was carried out between the participants’ judgment probability and the auditory ERP amplitude obtained when the somatosensory response was subtracted from “pair” responses and auditory-alone response. For the purpose of this analysis, both variables were transformed into z-scores in order to remove differences in amplitude variability between individuals. The analysis was applied independently at each electrode and for each response peak. Note that we did not apply correlation analysis to the data aligned at somatosensory onset because ERPs in each “pair” condition during a specific period relative to somatosensory onset represent different time periods in terms of auditory processing.

### BEHAVIORAL CONTROL

As a separate control, simultaneity judgments were obtained in order to determine whether participants perceived the temporal difference (simultaneous, 90 ms lead and lag) between somatosensory and auditory stimuli as simultaneous. We assessed the perceived temporal range of somatosensory onset relative to auditory onset when both stimuli were presented simultaneously. Six individuals participated in the test. The participants were presented with auditory and somatosensory stimulation and asked to report if stimuli were simultaneous or not. The test started with two initial values of somatosensory stimulation relative to auditory onset: (1) 300 ms lead and (2) 300 ms lag. The lead and lag conditions were alternated in random order. The temporal difference between the somatosensory and auditory stimulations was reduced based on participants’ response according to the Parameter Estimation by Sequential Testing (PEST) procedure (Macmillan and Creelman, 2004) until they reached a threshold level to detect the somatosensory–auditory stimulations as simultaneous or not.

## RESULTS

### SOMATOSENSORY-ALIGNED POTENTIALS

**FIGURE 2:**
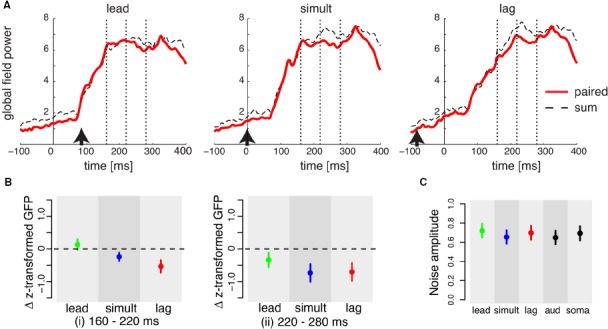
**(A)** Global field power (GFP) for somatosensory and auditory stimulation in the three timing conditions (lead, simultaneous, and lag). Each line represents the data averaged across all subjects. The vertical dotted lines define two intervals (i) 160–220 ms and (ii) 220–280 ms after somatosensory onset in which differences between “pair” and “sum” responses are assessed. Arrows represents auditory onset. **(B)** Difference in z-transformed GFP amplitude in the temporal periods of (i) and (ii) in panel **(A)**. Error bars give standard errors across participants. Each color represents a different experimental condition of relative timing difference. **(C)** GFP noise level in the period before the stimulation (-200 to -100 ms) in all conditions. Error bars represent the standard error across all subjects.

We first examined whether the timing difference between the sensory stimulation conditions induced changes in GFP. Figure 2A shows the GFP as the timing of stimulation varied (lead, lag, and simultaneous). The red thick line shows the GFP for the paired sensory condition and the black dashed line shows the sum of the two unisensory conditions (soma + aud). The data are aligned at somatosensory onset. The arrows represent the auditory onsets. The vertical dotted lines are the temporal intervals used to assess differences between conditions as a result of the timing of stimulation. Two empirically determined intervals were used to assess stimulus-timing effects after the end of the transient phase of the GFP change. The first interval is between 160 and 220ms after the somatosensory stimulus onset and the second is between 220 and 280ms. We found two pattern of differences in the target intervals respectively. For the first interval, the response amplitude difference between the “pair” and “sum” signals changed as a function of stimulus timing. In the lead condition, the “pair” GFP was marginally greater than the “sum” of the individual GFPs. The sign of the difference was reversed in the same and lag conditions. The difference in lag condition was greater than in the simultaneous condition. These amplitude differences are summarized in left panel of Figure 2B. Repeated measures ANOVA indicated reliable change across the three temporal conditions [*F*(2,22) = 7.76, *p* < 0.01]. For the second interval, the “pair” GFP was consistently smaller than the “sum” GFP regardless of the timing condition. These amplitude differences are summarized in the right panel of Figure 2B. Repeated measures ANOVA revealed that GFP in “pair” response was reliably smaller than GFP in the sum of unisensory responses [*F*(1,11) = 6.81, *p* < 0.03]. Note that there was a difference between sum and pair conditions before the transient phase (up to 80 ms after somatosensory onset). This is most likely due to an added effect of noise in the summed condition since this difference was not present for each component individually (see Figure 2C). Overall, these results suggest timing-related and timing-independent processing associated with separate stages of the evoked response.

**FIGURE 3:**
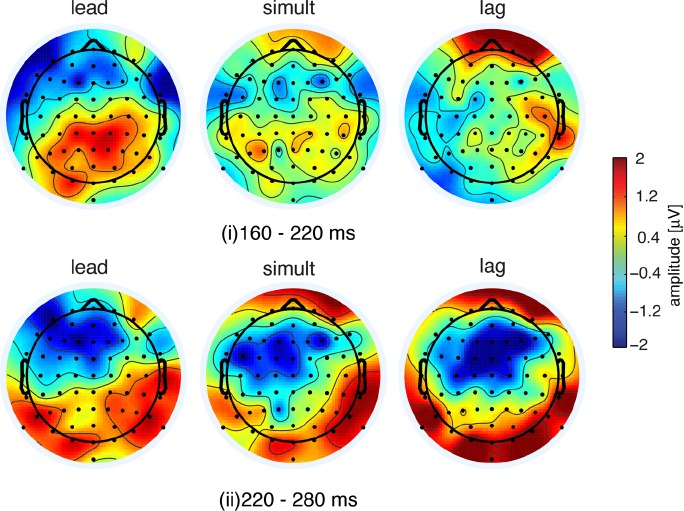
**Topographic maps of differences in event-related potentials between “pair” and “sum” conditions.** The top three panels represent the difference in the interval 160–220 ms and the bottom represents the difference at 220–280 ms after somatosensory onset.

We further investigated the trend observed in the GFP by examining the response patterns at individual electrodes. At the first target interval, that is, in the interval when somatosensory–auditory activation changed in a manner dependent on the relative timing of paired stimulation, the topographic configuration varied according to the relative timing of the stimulation (lead, simultaneous, and lag). Figure 3 shows topographic maps of the mean differences in amplitude between “pair” and “sum” responses. In the lead case, most of the differences were in the positive direction and were observed in the parietal electrodes. Similar differences in the parietal electrodes were also seen in the simultaneous condition, although the amplitude of the difference was smaller compared to the lead condition. In the lag condition, no difference was observed in the parietal electrodes, but several frontal electrodes showed a positive difference.

The similarity of the topographic configuration was assessed using global dissimilarity as a quantitative measure (DISS, review in Murray et al., 2008). DISS values indicated that the lead and lag conditions were topographically inverted (DISS = 1.41). On the other hand, lead and simultaneous conditions were moderately homogeneous (DISS = 0.82). The similarity between the simultaneous and lag conditions was not remarkable (DISS = 1.10), suggesting that the topography for the simultaneous condition was intermediate between the lead and lag conditions. The inverted topographic configuration between the lead and lag condition suggests that the topographic configuration was altered in conjunction with the timing differences between somatosensory and auditory stimulation onsets.

Event-related potential amplitude difference in the second target interval (220–280 ms after somatosensory onset) showed consistent change across the three “pair” condition in terms of GFP (Figure 2B, right panel). The topographic configuration during the period 220–280 ms was quite similar across three conditions (Figure 3, lower panels). We found that a broad range of frontal areas showed a reduction of “pair” responses in comparison to “sum” in all three temporal conditions, as was observed in GFP (Figure 2). Global dissimilarity for all three conditions is comparatively homogenous [DISS = 0.64 (lead and simultaneous), 0.49 (simultaneous and lag), and 0.70 (lead and lag)], suggesting that the amplitude reduction was present regardless of stimulus timing.

**FIGURE 4:**
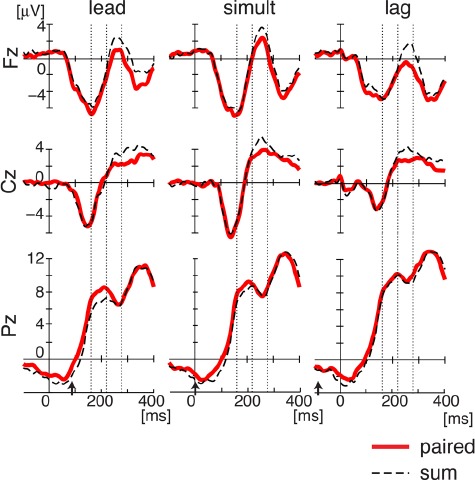
**Event-related potentials for combined somatosensory and auditory stimulation in the three timing conditions (lead, simultaneous, and lag) at Fz, Cz, and Pz.** Each line represents the data averaged across all subjects. Bias levels were adjusted using the average amplitude in the pre-stimulus interval (-200 to -100 ms). As in GFP analysis, the vertical dotted lines define two intervals (i) 160–220 ms and (ii) 220–280 ms after somatosensory onset in which differences between “pair” and “sum” responses are assessed. Arrows represents auditory onset.

Temporal patterns of ERP in representative electrodes (Fz, Cz, and Pz) are shown in Figure 4. As indicated in the GFP analysis, two patterns of change across three stimulus conditions were observed in the two temporal intervals respectively. In the first interval between 160 and 220 ms, repeated measure three-way ANOVA showed a reliable interaction effect across timing (lead, simultaneous, and lag), condition (pair and sum), and electrodes (Fz, Cz, and Pz) [*F*(4,44) = 3.175, *p* < 0.03]. In a more detailed analysis with Bonferroni correction, the difference in Pz amplitude between “pair” and “sum” ERPs changed as a function of the three stimulation conditions [*F*(2,22) = 5.99, *p* < 0.03], but there was no change and no difference in the other two electrodes [Fz: *F*(2,22) = 1.73, *p* > 0.6; Cz: *F*(2,22) = 1.00, *p* = 1.0]. In contrast, in the second interval between 220 and 280 ms there was a reliable interaction effect between experimental condition (pair and sum) and electrodes (Fz, Cz, and Pz) [*F*(2,22) = 9.812, *p* < 0.001]. Following Bonferroni correction, ERP amplitude at Fz and Cz in the “pair” condition was also consistently smaller than the “sum” ERP amplitude in the three stimulation conditions [Fz: *F*(1,11) = 8.32, *p* < 0.05; Cz: *F*(1,11) = 12.24, *p* < 0.02], but there was no difference at Pz [*F*(1,11) = 0.87, *p* = 1.0].

### AUDITORY-ALIGNED POTENTIALS

While we found a dynamical modulation of the ERP response in the context of somatosensory processing, it is difficult to interpret this change relative to speech processing since the observed modulation is not directly related to the timing of auditory processing. In order to compare paired auditory and somatosensory processing with that involved in speech perceptual processing, we examined these paired effects in relation to auditory-related processing on its own. We extracted auditory-related responses in the various paired conditions by subtracting the somatosensory-alone response from that obtained in the “pair” conditions. The logic is that if there is a non-linear interaction between somatosensory and auditory processing, the response after the subtraction should be different from the auditory alone response.

**FIGURE 5:**
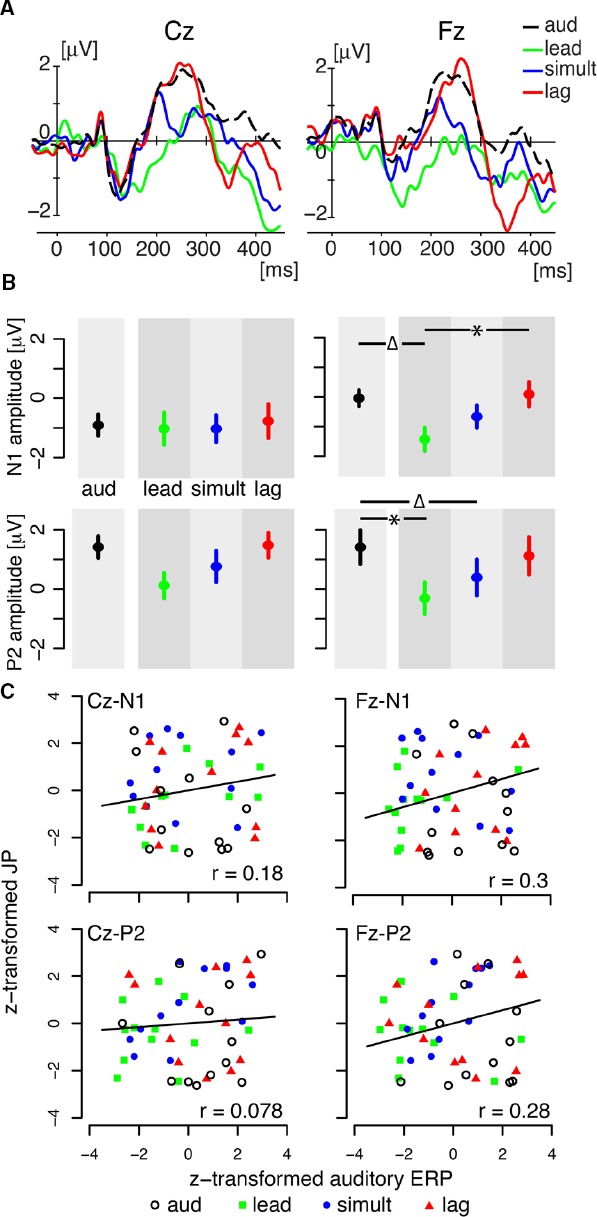
**(A)** Temporal responses of auditory event-related potentials at two mid-frontal electrodes (Fz and Cz). Each line represents the data averaged across all subjects. Each color corresponds to the following four conditions. “aud” represents the amplitude of auditory alone condition. “lead,” “simultaneous,” and “lag” represent the amplitudes in the corresponding three “pair” conditions after the removal of somatosensory potentials. Bias levels were adjusted using the average amplitude in the pre-stimulus interval (-200 to -100 ms). **(B)** Peak amplitude of N1 and P2 auditory event-related potentials at two mid-frontal electrodes. The left two panels are for Cz, and the right two panels for Fz. Error bars give standard errors across participants. The symbol “*” represents reliable difference (*p* < 0.05) and “Δ” represents marginal difference (*p* ≤ 0.1). **(C)** Correlation plots between peak amplitude of ERP and judgment probability for the behavioral task (48 data points from 12 subjects × 4 conditions).

For this analysis, all of the data were aligned at auditory onset. The subtracted potentials and the auditory-alone potentials showed a typical N1–P2 pattern with the first negative peak (N1) between 100 and 200 ms after auditory onset followed by a second positive peak (P2) between 200 and 300 ms (see Figure 5A). The maximum response was observed along mid-line electrodes near Cz (vertex electrode).

The peak amplitude at the Cz and Fz electrodes was quantified using 60-ms temporal window in each of the three “pair” timing conditions (lead, simultaneous, and lag) and for the auditory response alone (Figure 5B). Each color represents a different condition. Error bars represent the standard error across participants. Two way-repeated measure ANOVA (timing condition × electrodes) yielded reliable differences across timing condition (lead, lag, and simultaneous) in N1 [*F*(3,77) = 3.056, *p* < 0.05] and in P2 [*F*(3,77) = 6.18, *p* < 0.001]. By looking at ERPs in each individual electrode, we found a consistent N1 response at Cz in all four conditions (lead, simultaneous, lag, and auditory). The peak amplitudes were not statistically different for the four conditions [*F*(3,33) = 0.122, *p* > 0.9]. The peak amplitude of the P2 response showed a graded change according to the stimulus timing (lead, simultaneous, and lag), although the change was statistically marginal as follows. Whereas repeated measures one-way ANOVA showed reliable difference across the four conditions [*F*(3,33) = 3.82, *p* < 0.04], *post hoc* testing did not reveal any reliable paired comparisons.

In contrast, a reliable change was observed at Fz electrode in both N1 and P2 amplitude (see right two panels in Figure 5B). N1 responses at Fz were reliably different across the four conditions [*F*(3,33) = 5.95, *p* < 0.02]. *Post hoc* tests with Bonferroni correction showed that the lead condition was reliably different from lag condition (*p* < 0.02) and marginally different from the auditory response (*p* = 0.06). P2 response were also reliably different across conditions [*F*(3,33) = 4.80, *p* < 0.02]. Comparing the auditory alone responses with the other “pair” condition yielded a reliable difference from the lead condition (*p* < 0.05) and a marginal difference from simultaneous condition (*p* = 0.10). The difference for the lag condition was not reliable (*p* > 0.9). Overall, the results reveal that auditory ERPs show a change when combined with temporally offset somatosensory stimulation. The largest change occurs when somatosensory stimulation lead for the speech sound. On the other hand, when somatosensory stimulation lags speech onset, the amplitude of the auditory potentials are no different from the potentials for auditory stimulation alone.

### BEHAVIORAL PERFORMANCE

We also examined the behavioral results and their relationship with EEG activity. There was no reliable change of judgment probability in the three paired conditions in comparison to the auditory alone condition [*F*(3,33) = 1.128, *p* > 0.3]. Correlation analysis showed that the judgment probabilities in the four conditions were reliably correlated with N1 amplitude at Fz (*r* = 0.3, *p* < 0.05) and marginally correlated with P2 amplitude at Fz (*r* = 0.28, *p* = 0.05). The peak amplitude of N1 and P2 at Cz were not reliably correlated with the judgment probabilities (N1: *r* = 0.18, *p* > 0.2; P2: *r* = 0.078, *p* > 0.6). Figure 5C shows the correlation plots in each combination of N1 and P2 responses at Cz and Pz. Thus, overall, although the magnitude of the correlation was relatively low, the results suggest that perceptual modulation as measured behaviorally may be represented to some degree in the cortical response at Fz.

Reaction times across the five conditions: three “pair” conditions and two uni-sensory conditions “soma” and “aud” were evaluated. We did not find any reliable differences across all five conditions [*F*(4,44) = 0.532, *p* > 0.70]. This is inconsistent with typical responses due to multisensory stimulation conditions. Reaction time to respond to stimuli typically becomes shorter when two sensory modalities were stimulated simultaneously than when single sensory modalities are stimulated. The difference from the typical multisensory reaction may presumably be because the current task involved identification only.

### BEHAVIORAL CONTROL

In order to examine if the time differences between stimuli in the “pair” condition are perceived as simultaneous, we obtained threshold values at which the participants determined whether or not the somatosensory and auditory stimulations were simultaneous. The average threshold times for perception of simultaneity were 210.6±3.1 ms lead and 148.0±3.9 lag of the somatosensory onset relative to the auditory onset (see rectangle and error bar in Figure 1A). The results indicates that the 90 ms difference used in the current ERP recording is in a range where stimuli are perceived to be simultaneous and suggests the participants did not perceive a difference in stimulus timing in any of the three “pair” conditions.

## DISCUSSION

This study assessed the neural correlate of the temporal interaction between orofacial somatosensory and speech sound processing. The cortical activity associated with orofacial somatosensory–auditory interaction was quantified using ERPs. We found two types of non-linear interactions between somatosensory and auditory processing. One form of sensory interaction was dependent on the relative timing of the two sensory stimuli. The other was constant regardless of stimulus timing. The two interactions were observed at different electrodes sites: the stimulus timing interaction was recorded over parietal electrodes and the non-stimulus timing interaction was observed over the frontal electrodes. From the viewpoint of auditory processing, we also found a graded change in ERP amplitudes that was dependent on the relative timing of stimuli for auditory processing. The results demonstrate clear multisensory convergence and suggest a dynamic modulation of these particular (somatosensory–auditory) interactions during speech processing.

It is important to note that in the previous psychophysical study demonstrating an interaction between speech sound processing and orofacial stimulation, perceptual judgments were influenced by speech-production-like patterns of facial skin stretch (Ito et al., 2009). The current finding showing the largest amplitude change in the multisensory evoked response occurring with a somatosensory lead is also consistent with speech production-like patterning affecting cortical evoked potentials. Auditory input from self-generated speech is always preceded by articulatory motion that generates somatosensory input in advance of the acoustic signal. Interestingly, Möttönen et al. (2005) showed that simple lip tapping during speech perceptual processing did not change magnetoenchalographic evoked potentials. It appears, consistent with the previous psychophysical study (Ito et al., 2009), that the influence of somatosensory stimulation on speech perceptual processing may be dependent on a functional relationship between the somatosensory characteristics of the stimulation and those accompanying speech production. Hence, somatosensory inputs that are similar to those experienced in speech production can interact effectively with speech sound processing and the interaction is reflected in cortical potential changes.

The timing of sensory stimulation is a key factor in multisensory interaction. The effective time-window for multisensory integration is known to be as long as 200 ms (Meredith et al., 1987; van Wassenhove et al., 2007). At a behavioral level, this is consistent with the results of our control test in which the participants perceived the skin stretch perturbation and the speech sound “*head*” as simultaneous in a comparable temporal range. Although the neural correlates of AV interaction including that involving speech stimuli has been previously investigated (Pilling, 2009; Vroomen and Stekelenburg, 2010; Liu et al., 2011), the temporal range was larger than 200 ms, and hence it is not known the extent to which multisensory interactions occur at shorter temporal asynchronies. In the present study, dynamical modulation at an electrocortical level was found at a range of 100 ms. The current finding suggests cortical processing is sensitive to temporal factors even within the time range at which events are behaviorally judged simultaneous.

In AV speech, the effective temporal range between auditory and visual stimulus onsets for effective multisensory interaction is asymmetric in terms of onset timing. While AV speech phenomena, such as the McGurk effect is induced with up to a 240-ms of visual lead, while for visual lag the time window is much shorter (up to 40 ms; Munhall et al., 1996; van Wassenhove et al., 2007). Our ERP findings may be comparable. N1 and P2 potential amplitudes in the 90 ms somatosensory lag relative to auditory onset were not different from those in the auditory alone response, whereas the lead and simultaneous condition showed a difference between the “pair” and “sum” responses, indicating that the somatosensory lead condition has affected audio processing, but not in the lag condition. This can probably be attributed to the temporal relationship between orofacial somatosensory inputs and acoustic output in speech production, since articulatory motion mostly precedes acoustic output in speech production (e.g., Mooshammer et al., 2012).

In addition to the differential cortical response dependent on the asynchrony of the somatosensory–auditory stimulation, we also found a consistent reduction in the cortical response independent of the asynchrony of the stimulation in the later period (220–280 ms after somatosensory onset). Interestingly this reduction was seen only in the somatosensory analysis suggesting that this later period of somatosensory processing consistently interacts with the auditory input regardless of the timing of auditory processing. While such an obligatory multisensory interaction is a plausible interpretation, the reduction could be influenced by non-stimulus-specific factors such as changing attentional demands. However, the use of stimulus averaging over a number of the responses time-locked to the onset of specific stimulus would most likely eliminate or minimize any non-stimulus-specific effects. Consequently, the possibility of a non-specific effect to explain the consistent reduction in the cortical response is unlikely.

Two different patterns of activation were observed depending on the asynchrony of the stimulation and the specific time post-stimulation onset. The asynchrony dependent modulation was observed in a period between 160–220 ms after somatosensory onset mostly at parietal electrodes. In contrast, frontal electrodes showed a consistent multisensory change in activation in all three temporal conditions during the 220–280 ms period after somatosensory onset. Since multiple subcortical and cortical locations are involved in auditory–somatosensory interactions in non-speech processing (Stein and Meredith, 1993; Foxe et al., 2002; Lütkenhöner et al., 2002; Fu et al., 2003; Kayser et al., 2005; Murray et al., 2005; Schürmann et al., 2006; Shore and Zhou, 2006; Lakatos et al., 2007; Beauchamp et al., 2008), the present results reflect the contribution of different and distributed cortical sites in the somatosensory–auditory interaction during speech processing. Given that the parietal area and planum temporale is considered as a center of auditory–motor integration (Hickok et al., 2011; Tremblay et al., 2013), the parietal site may also be important for the temporal processing between somatosensory and auditory inputs.

While we found a reliable difference for the timing manipulation in the ERP changes, there was no such reliable result in the behavioral measure. It appears that the nervous system is sensitive to timing differences in the relatively early phase of speech processing (N1 and P2), but this difference may be independent of the ability to identify such differences. This is not surprising given the additional cognitive process involved in perceptual judgments. It appears that a more sensitive task is required to detect these subtle timing differences behaviorally. The current experimental design was optimized for identifying the influence of sensory input on cortical potentials rather than on cognitive decisions.

A possible neural pathway for somatosensory influence on speech perception is currently unknown. For language processing, the posterior inferior frontal gyrus (Broca’s area) is known to contribute to speech perception, and hence the neural connections between the prefrontal and the temporal areas associated with auditory processing have been well documented (Geschwind, 1970; Margulies and Petrides, 2013). Given a connection between the premotor and somatosensory cortex, and the premotor cortex and Broca’s region (Margulies and Petrides, 2013), somatosensory inputs may influence auditory processing via the prefrontal gyrus and the premotor cortex. On the other hand, studies of non-speech processing have shown that somatosensory inputs directly affect lower levels of auditory processing in the auditory cortex and/or the surrounding areas (Foxe et al., 2002; Lütkenhöner et al., 2002; Fu et al., 2003; Kayser et al., 2005; Murray et al., 2005; Schürmann et al., 2006; Lakatos et al., 2007; Beauchamp et al., 2008). These non-speech studies have shown a reciprocal somatosensory–auditory interaction that is independent of motor involvement, in particular, change in second somatosensory cortex in response to sound and changes in auditory cortex in response to somatosensory stimulation. In addition, there are somatosensory–auditory interactions in subcortical areas (Stein and Meredith, 1993; Shore and Zhou, 2006). These results suggest a tight linkage and direct neural connection between somatosensory and auditory system that is separate from a motor–auditory connection mentioned above. The current findings show a dynamic modulation of the effects of somatosensory and auditory stimuli at the electrodes over the parietal region. This is consistent with the idea of direct access of somatosensory inputs to the auditory system. Since speech and non-speech sounds are processed differently in the brain (Kozou et al., 2005; Möttönen et al., 2006), it is unclear whether pathways associated with somatosensory–auditory interactions in non-speech processes are also involved in speech processing. Further investigation is required.

The linkage between speech production and perception processing has been a topic of interest for over five decades (Liberman et al., 1967; Diehl et al., 2004; Schwartz et al., 2012). Whereas the idea has been previously tested from the viewpoint of speech production and motor function (Fadiga et al., 2002; Watkins et al., 2003; Wilson et al., 2004; Meister et al., 2007; D’Ausilio et al., 2009; Möttönen and Watkins, 2009), the role of somatosensory function in speech perception has been overlooked. Previous psychophysical findings have showed that orofacial somatosensory inputs can influence speech processing (Ito et al., 2009). The current findings further suggest that somatosensory stimulation has access to cortical areas associated with speech processing. One intriguing possibility is that somatosensory information may be an important component in establishing the neural representations for both speech production and speech perception. Further investigation of the manner in which orofacial somatosensation modulates speech perceptual processing may provide some important clues to understanding the development of the linkage between speech perception and production.

### Conflict of Interest Statement

The authors declare that the research was conducted in the absence of any commercial or financial relationships that could be construed as a potential conflict of interest.
